# 3D Printing Hierarchically Nano‐Ordered Structures

**DOI:** 10.1002/advs.202302756

**Published:** 2023-08-02

**Authors:** Britta Weidinger, Guohui Yang, Nadine von Coelln, Hermann Nirschl, Irene Wacker, Petra Tegeder, Rasmus R. Schröder, Eva Blasco

**Affiliations:** ^1^ Insitute for Molecular Systems Engineering and Advanced Materials Universität Heidelberg Im Neuenheimer Feld 225 69120 Heidelberg Germany; ^2^ Institute of Organic Chemistry Universität Heidelberg Im Neuenheimer Feld 270 69120 Heidelberg Germany; ^3^ Institute of Mechanical Process Engineering and Mechanics, Karlsruhe Institute of Technology (KIT) 76131 Karlsruhe Germany; ^4^ Physikalisch‐Chemisches Institut Universität Heidelberg Im Neuenheimer Feld 253 69120 Heidelberg Germany; ^5^ BioQuant Universität Heidelberg Im Neuenheimer Feld 267 69120 Heidelberg Germany

**Keywords:** 3D printing, block polymers, direct laser writing, self‐assembly, two‐photon polymerization

## Abstract

Natural materials are composed of a limited number of molecular building blocks and their exceptional properties are governed by their hierarchical structure. However, this level of precision is unattainable with current state‐of‐the‐art materials for 3D printing. Herein, new self‐assembled printable materials based on block copolymers (BCPs) enabling precise control of the nanostructure in 3D are presented. In particular, well‐defined BCPs consisting of poly(styrene) (PS) and a polymethacrylate‐based copolymer decorated with printable units are selected as suitable self‐assembled materials and synthesized using controlled radical polymerization. The synthesized library of BCPs are utilized as printable formulations for the fabrication of complex 3D microstructures using two‐photon laser printing. By fine‐tuning the BCP composition and solvent in the formulations, the fabrication of precise 3D nano‐ordered structures is demonstrated for the first time. A key point of this work is the achievement of controlled nano‐order within the entire 3D structures. Thus, imaging of the cross‐sections of the 3D printed samples is performed, enabling the visualization also from the inside. The presented versatile approach is expected to create new avenues for the precise design of functional polymer materials suitable for high‐resolution 3D printing exhibiting tailor‐made nanostructures.

## Graphical abstract

Herein, a novel printable material suitable for two‐photon laser printing based on self‐assembled block copolymers is presented. The creation of 3D structures entailing high resolution, complex geometry, and a controlled morphology, such as lamella und cylindric nanostructures, is successfully demonstrated.

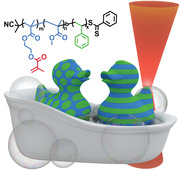

## 1 Introduction

Additive manufacturing (3D printing) has gained much attention during the last years, becoming a promising fabrication tool in a wide range of size regimes (from nano‐ to macroscale) and application fields, varying from biomedicine to optics or aerospace, among others.^[^ ^1^, ^2^, ^3^ ^]^ For many applications, high precision and accuracy during fabrication are crucial. Direct laser writing (DLW), also known as two‐photon laser printing (TPLP), has become a key technology enabling the precise fabrication of structures at the microscale with sub‐micron resolution.^[^ ^4^, ^5^, ^6^, ^7^, ^8^, ^9^ ^]^ This technique relies on multi‐photon polymerization of a photoreactive ink. Concerning the materials, commercially available acrylate‐based as well as epoxy‐based photoresists have been widely employed.^[^ ^10^ ^]^ Recently, considerable efforts have been made to expand the possibilities of this technology by designing new materials incorporating different properties such as conductivity,^[^ ^11^ ^]^ biocompatibility,^[^ ^12^ ^]^ or adaptivity.^[^ ^13^ ^]^ However, there is still a long way to go to reach the level of precision and functionality of natural systems.

Natural materials are comprised of a limited number of molecular building blocks (e.g., amino acids, carbohydrates). However, their exceptional performance is the result of hierarchical order on multiple length scales.^[^ ^14^, ^15^ ^]^ This perfection in the structure‐function relationship is the result of evolution over billions of years. Investigation of self‐assembled synthetic materials has been the focus of many researchers within the last decades.^[^ ^16^, ^17^, ^18^ ^]^ Recently, this concept is also emerging for printable materials, in particular for light‐based 3D printing.^[^ ^19^ ^]^ For example, several groups have exploited the use of photoinitiated polymerization‐induced microphase separation for the preparation of hierarchical, inherently porous 3D objects using digital light processing (DLP) as the 3D printing method.^[^ ^20^, ^21^ ^]^ There, phase separation between polymer and porogen (e.g., organic solvents) occurs upon printing and therefore, forming a porous 3D structure, whose pore size can be tuned with the printing formulation. More recently, Boyer and co‐workers used a similar principle to realize macroscopic objects exhibiting different nanoscale morphological features.^[^ ^22^, ^23^ ^]^ In this case, polymerization‐induced microphase separation utilizing reversible‐deactivation radical polymerization led to in‐situ block copolymer (BCP) formation during 3D printing, which resulted in materials that displayed a variety of nanoscale domains. This methodology enables remarkable nanoscale control in 3D printed structures. However, as the self‐assembly takes place during the printing process, the polymer network is formed in a kinetically trapped state, leading to fixed morphologies that are not in thermodynamic equilibrium. Therefore, a long‐range order in 3D cannot be achieved, limiting the potential applications.

**Figure 1:**
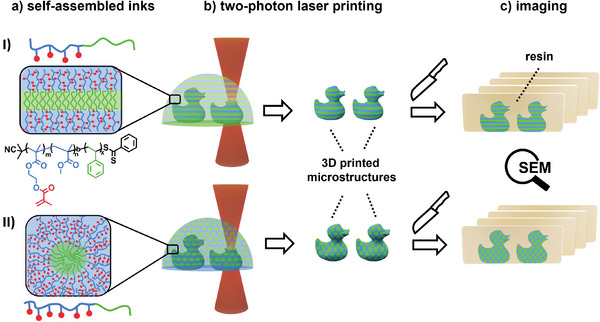
Schematic illustration of the overall approach. a) Pre‐assembled functional BCP based inks exhibiting lamellar (I) or cylindrical morphology (II) are utilized for b) the fabrication of defined 3D structures exhibiting nano‐order using TPLP. c) For characterization, the 3D printed microstructures are embedded into epoxide resin, sectioned using an ultramicrotome, and the internal nanostructure is imaged via SEM. Note: the nanodomains of lamella and cylindrical structures are “oversized” for illustrative purposes.

Herein, we present a novel approach allowing for the creation of 3D hierarchical ordered structures by combining self‐assembled block copolymer‐based inks and TPLP (**Figure** **1**). Self‐assembled BCPs exhibiting high‐ordered nanostructures have been extensively investigated in 2D films,^[^ ^24^, ^25^ ^]^ but have not yet been exploited to generate 3D structures that entail high resolution, complex geometry, and a controlled nanostructure. Thus, a key aspect of this work is the utilization of inks consisting of pre‐self‐assembled defined block copolymers. To achieve this aim, well‐defined BCPs consisting of a poly(styrene) (PS) block and a polymethacrylate‐based block were synthesized and post‐modified to introduce the printable moieties, i.e., methacrylates. The functional BCPs are formulated into self‐assembled inks using a photo‐initiator and a suitable solvent. TPLP is employed to fix the nanostructure while creating a defined 3D architecture. The self‐assembly behavior of the synthesized BCPs is characterized in depth in every stage: 1) prior to printing (in the self‐assembled ink), 2) in bulk polymer films, and 3) after printing. Furthermore, cross‐sections of the 3D printed microstructures were imaged using scanning electron microscopy (SEM) showing highly ordered nanostructures for the first time.

## 2 Results and Discussion

### 2.1 Designing Printable Self‐Assembled Materials based on BCPs

When designing the printable system, the following requirements were considered: 1) phase separation at the nanoscale and 2) light reactivity to ensure printability using two‐photon laser printing. As stated above, BCPs have been selected as ideal building blocks for the design of this new generation of printable inks. To meet the first criterion, BCPs consisting of a polystyrene (PS) block and a polymethylmethacrylate (PMMA) based block were chosen, since PS‐*b*‐PMMA is a well‐studied system in terms of self‐assembly.^[^ ^26^, ^27^, ^28^ ^]^ Secondly, by introducing a functional methacrylate co‐monomer in the PMMA block, hydroxyethyl methacrylate (HEMA) in this case, a post‐modification route is possible, which is key for the anchoring of photo‐cross‐linkable groups for printing.

**Figure 2:**
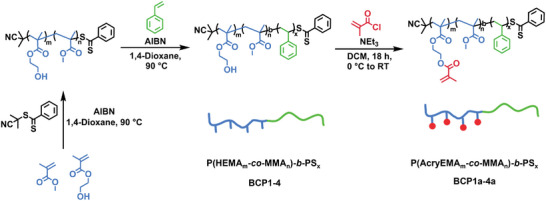
Synthetic strategy for the preparation of 3D‐printable BCPs via sequential RAFT‐controlled radical polymerization followed by incorporation of methacrylate groups. Exact compositions are indicated in Table 1.

To synthesize the targeted BCPs, sequential RAFT (reversible addition‐fragmentation chain transfer) polymerization, which is characterized by its high tolerance to functional groups, and a relatively high tolerance to impurities, was employed (**Figure** **2**). First, MMA and HEMA were copolymerized with cyanopropyldithiobenzoate (CPDB) as the chain transfer agent (CTA) to prepare several macroCTAs (1‐4) with different MMA/HEMA ratios and molecular weights (Table S1, Supporting Information). Afterward, the macroCTAs were chain extended with styrene to yield a library of BCPs having hydroxy groups as side chains in the methacrylic block. To introduce the cross‐linkable moiety, this free hydroxy group was esterified using methacryloyl chloride. An excess of methacryloyl chloride was employed to ensure full conversion, which was monitored via ^1^H‐NMR. By following this strategy, we were able to synthesize a library of BCPs with a broad range of compositions and molecular weights, and a selection of these is shown in **Table** **1**. First, a nearly symmetrical block copolymer (BCP1) with a molar ratio of MMA/HEMA to styrene of 47:53 was synthesized to target a lamellar morphology. Afterward, the fraction of the MMA/HEMA block was varied for BCP2 and BCP3, with respective MMA/HEMA contents of 63% and 34% aiming at other complex morphologies such as gyroids or cylinders. By inverting the major component, the matrix surrounding, e.g., the cylinders should be switched (from PMMA‐based to PS‐based). For BCP4, the HEMA‐fraction in the methacrylate blocks was increased from previously 22% to 46% to investigate the influence on self‐assembly as well as printability. All synthesized BCPs (before and after functionalization with photocrosslinkable groups) were characterized by nuclear magnetic resonance (NMR) spectroscopy and gel permeation chromatography (GPC) (see Table 1 and Figures S1– S6, Supporting Information), enabling the determination of the exact composition and relative molecular weight. Importantly, all the BCPs showed low dispersities and monomodal GPC curves.

**Table 1:** Composition, molecular weight, dispersity, and molar fraction of the synthesized BCPs

Polymer	M_n_ [GPC]^a^ ^)^	Đ	f_MMA‐HEMA :_ f_styrene_ [mol%]^#^ ^)^	Modified Polymer
BCP1 P(HEMA_93_‐MMA_329_)‐*b*‐PS_323_	93566	1.10	47: 53	BCP1a P(AcryEMA_93_‐MMA_329_)‐*b*‐PS_323_
BCP2 P(HEMA_85_‐MMA_301_)‐*b*‐PS_229_	65036	1.09	63: 37	BCP2a P(AcryEMA_85_‐MMA_301_)‐*b*‐PS_229_
BCP3 P(HEMA_50_‐MMA_176_)‐*b*‐PS_437_	69535	1.13	34: 66	BCP3a P(AcryEMA_50_‐MMA_176_)‐*b*‐PS_437_
BCP4 P(HEMA_138_‐MMA_162_)‐*b*‐PS_199_	54974	1.15	60: 40	BCP4a P(AcryEMA_138_‐MMA_162_)‐*b*‐PS_199_

^a)^ eluent THF, calibrated against PMMA standards;

^#)^ calculated from ^1^H NMR.

As a next step, and prior to the printing experiments, the self‐assembly properties of the BCPs in bulk before and after functionalization with cross‐linkable groups, *i.e*., methacrylate moieties, were investigated. To this aim, films were cast from chloroform by slow evaporation of the solvent. The resulting thick films were then embedded, sectioned, stained, and analyzed by SEM, providing insights into the morphology of the bulk material. Additionally, the films were directly analyzed by small angle X‐ray scattering (SAXS), and further insight into the sections was gained by imaging with infrared scanning nearfield optical microscopy (SNOM).

**Figure 3:**
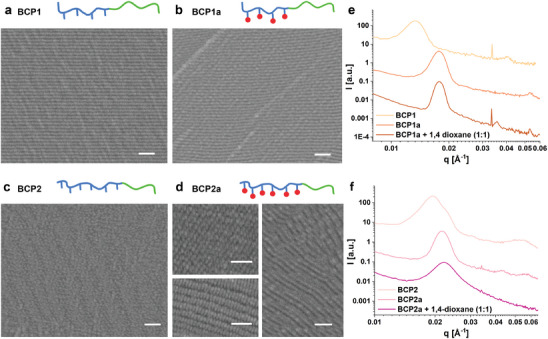
Microscale analysis of self‐assembled films: SEM images of cross‐sectioned films, showing the bulk self‐assembly of a) BCP1 (lamellae), b) BCP1a (lamellae), c) BCP2 (gyroid nanostructure), d) BCP2a (cylindrical nanostructure in different planes, see SI for further information). Scale bar = 200 nm. SAXS pattern of the films and concentrated solutions for e) BCP1(a) and f) BCP2(a). Spectrum intensities are offset for clearer visual comparison.

The SEM analysis of the bulk cross‐sections of all the polymers (BCP1(a) – BCP4(a)) showed clear phase segregation (**Figure** **3**a–d and Figure S7, Supporting Information). The nearly symmetrical BCP1 showed the expected lamellar nanostructure with long‐range order over several micrometers (Figure 3a). This highly ordered lamellar structure was also confirmed by SAXS measurements (shown in Figure 3e and Figure S8, Supporting Information), where the patterns exhibited peaks with a ratio of their associated *q*‐values of q* and 3q*. 2q* is not strongly exhibited, indicating that both blocks have the same size.^[^ ^29^ ^]^ Calculated from *d* = 2pi/*q*,^[^ ^30^ ^]^ this corresponds to lamellae with a domain spacing of 47 nm, which is comparable with domain sizes of around 41 nm measured from SEM images. In the case of the functionalized BCP1a, a clear lamellar nanostructure was also observed by SEM and SNOM (Figure 3b and Figure S9, Supporting Information). The SAXS pattern of BCP1a showed a shift of *q* to a higher value, indicating a smaller domain spacing of 35 nm, in accordance with the 36 nm domain spacing measured from the SEM image (Figure 3b). For BCP2, having a higher MMA/HEMA:styrene ratio, a morphology that can be assigned to gyroid structures was observed. The main indication is the characteristic shoulder at √(4/3) q* represented in the SAXS pattern (Figure 3f).^[^ ^31^ ^]^ Interestingly, when this BCP is functionalized to BCP2a, a cylindrical morphology is detected, confirmed by the SEM images in combination with the diffraction pattern and the SNOM imaging (Figure 3d and Figure S9, Supporting Information). We believe that this shift in the morphology after introduction of the functional group is due to the change in weight/volume fractions and interaction parameters c. As before, *q* increased upon introduction of the functional group, and the corresponding decrease in domain size is also measurable in the SEM images. Due to the 3‐dimensionality of the bulk film, there is little control over which orientation the morphology is intersected. Figure 3d shows cut through several planes of the self‐assembled cylinders, and the corresponding 3D modeled renderings can be found in Figure S10 (Supporting Information). In the case of BCP3 and BCP4, and the corresponding functionalized polymers, a clear switch from lamellae to cylindrical morphology was observed (see Figure S7, Supporting Information).

### 2.2 Ink Formulation and 3D Printing of Hierarchically Ordered Structures

As a next step, the printability of the self‐assembled materials was examined. In this step, the pre‐formed morphologies are fixed via the photo‐crosslinking of the material using the laser, while being shaped into a 3D geometry. The key challenge of this work was to ensure good printability of the self‐assembled material while preserving the nano‐order during the printing process and importantly, within the entire 3D structure. Printing directly the bulk self‐assembled, solid film was not possible, presumably due to laser heating. Therefore, the use of an ink formulation containing solvent was essential. Based on previous publications,^[^ ^32^, ^33^, ^34^, ^35^ ^]^ where self‐assembly was demonstrated for BCPs in highly concentrated, this possibility was investigated for our printable BCPs. As chloroform was successfully used for the preparation of the bulk films, which exhibited good self‐assembly behavior, this solvent was the first choice. However, its low boiling point led to evaporation during the ink preparation and printing process. Therefore, it was excluded due to the lack of reproducibility. Next, DMAc was tested as a potential candidate since it has a high boiling point (avoiding evaporation and concentration changes during printing) and the synthesized BCPs are well soluble in it. However, the SAXS measurements indicated a low degree of self‐organization, showing broad and weak peaks (Figure S11, Supporting Information). Hansen solubility parameters (see Table S2, Supporting Information) show that DMAc is more selective for the methacrylate‐based block as opposed to the styrene block, which is not suitable for this purpose. As a next step, tetrahydrofuran (THF) and 1–4 dioxane were considered, which both have Hansen solubility parameters that are close for these two polymer classes. For both solvents, the SAXS measurements confirmed the self‐assembly of the BCP‐based inks. The SAXS patterns of BCP1a in 1,4‐dioxane showed a similar diffraction pattern as well as a comparable position of *q* as the film of BCP1a (Figure 3e). Similar results were obtained for BCP2a (Figure 3f). Besides, the SAXS pattern from BCP1a in THF indicated lamellar organization (Figure S11, Supporting Information), confirming the successful self‐assembly in the concentrated solution of both, THF and 1,4‐dioxane.

**Figure 4:**
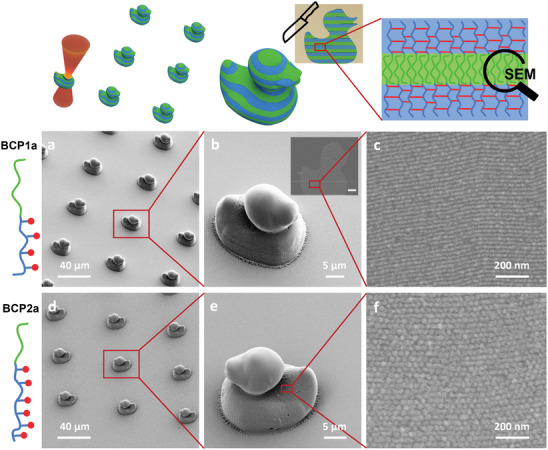
SEM characterization of 3D printed microstructures from a–c) BCP1a or d–f) BCP2a. Overall morphology (a,b and d,e) and cross sections prepared from resin‐embedded microstructures (insets in b). High‐resolution SEM images of cross sections (c,f) show lamella for BCP1a (c) and cylinders for BCP2a (f). Images of larger areas can be found in Figures S13 and S14, (Supporting Information). The microstructures were sectioned perpendicular to the substrate as illustrated by the inset in (b).

Once the self‐assembly of the BCPs in THF and 1,4‐dioxane was confirmed, the printability was investigated using a commercial two‐photon laser printer (Photonic Professional GT2 from Nanoscribe GmbH). In particular, ink formulations consisting of 50 wt.% functionalized polymer (BCP1a‐4a) in solvent and DETC as an efficient multi‐photon photoinitiator (0.25 wt.%) were employed for the printing test. First, the printing parameters (laser power, scanning speed, hatching, and slicing) were carefully optimized by printing arrays of the geometries of interest with systematically changing parameters so that fully solid structures could be printed without microbubble formation and loss of details while having a solid surface without visible print lines. The suitable dose was structure dependent, with laser powers ranging from 17.5 to 22.5 mW with scanning speeds between 2000 and 2500 µm s^−1^. In order to prove the versatility of the approach, the fabrication of different shapes with different complexity, e.g. pillars, octopi, and a half cylinder (Figure S12, Supporting Information) and micrometric rubber ducks with a size of 18 × 25 × 26 µm^3^ (**Figure** **4**) were successfully 3D printed.

After development, SEM characterization was performed to assess the printing quality. Using THF as the solvent in the ink, the 3D printed microstructures frequently showed defects in the top layer (Figure S15, Supporting Information), probably due to the rapid evaporation of the residual THF after development of the structures. However, for the formulations containing 1,4‐dioxane, SEM analysis of the printed structures showed higher quality of the 3D printed microstructure, as shown in Figure 4a,b,d,e. Comparing the different BCPs, BCP1a and 2a exhibited good printability, while the performance of BCP3a was worse, most probably due to the lower functionalization degree with methacrylate groups. BCP4a – having a higher functionalization degree – was printable comparable to BCP1a and BCP2a, but the lower solubility in 1,4‐dioxane was problematic in terms of obtaining a homogenous solution when performing the chain extension of macroCTA3. These results indicate the importance of the polymer design not only in terms of the block fraction ratio to ensure the BCP self‐assembly, but also in the incorporation of a suitable number of photo‐cross‐linkable groups to ensure good solubility. Thus, the in‐depth analysis of the self‐assembly of the 3D printed samples was focussed on the microstructures from BCP1a and 2a, which exhibited different morphologies in the bulk films.

The morphology inside the 3D printed microstructure was analyzed in cross‐sections of the printed structures. For this, the 3D‐printed microstructures were embedded into resin, sectioned by ultramicrotomy, and imaged by SEM after selective staining to introduce contrast between the PS and the methacrylate‐based blocks. This part was crucial to validate the retention of the pre‐formed nanostructure after printing and is the first example of using this type of analysis for this kind of printable BCP materials. As mentioned above, due to the better quality of the structures using 1,4‐dioxane as solvent, a more detailed analysis of 3D printed microstructures using this solvent was carried out. SEM images of the stained cross‐sections confirmed the morphology already prominent in the bulk polymer and in the SAXS measurements of the ink. Highly ordered lamellae (for BCP1a) and cylinders (for BCP2a) are clearly visible in the SEM images of the cross‐section of the 3D micrometric ducks (Figure 4c,f). For the lamellae, the order is especially remarkable. These lamellae are parallel without any junctions or other defects over areas of several µm^2^. Importantly, the domain spacing measured from the images is 36 nm and thus in agreement with the distances calculated from the X‐ray scattering pattern of the ink. This clearly indicates that we are able to retain the morphology from the bulk polymer and the ink mixture during the 3D printing process and development, and more importantly, the nanostructures are also present in the inside (bulk) of the 3D structure.

To verify that the visible nanostructure is an inherent property of the self‐assembly of the printable materials and not a printing artifact stemming from the printing of a higher molecular weight polymer in solution, we additionally prepared control samples in the solvents used for printing containing only the polymethacrylate‐based block containing photo‐cross‐linkable groups. The SEM images (see Figure S16, Supporting Information) of these cross‐sectioned samples do not show any nanostructure, further proving that the hierarchical structures can only be achieved by BCP self‐assembly.

## 3 Conclusion

We have presented a novel ink suitable for high‐resolution two‐photon 3D printing based on self‐assembled BCPs containing photo‐cross‐linkable groups. To this aim, a library of BPCs having different compositions and degrees of functionalization has been synthesized using controlled radical polymerization and carefully characterized. Furthermore, it was demonstrated that the self‐assembly and printability of these BCPs are strongly dependent on the solvent used. Highly concentrated solutions of BCP1a and BCP2a in 1,4 dioxane have shown good performance enabling the fabrication of complex 3D structures. Cross‐sections of the 3D printed samples were imaged using SEM, showing highly ordered and defined nanostructures such as lamella and cylindric morphologies, depending on the BCP composition. Although this work has been focussed on PS‐*b*‐PMMA‐based polymers, the presented approach is very versatile and can be easily expanded to other functional BCPs. By modifying the macromolecular design, e.g., length of the blocks, sequence, and composition, new nanostructures and functionalities can also potentially be obtained. For example, incorporation of responsive monomers will enable the careful control of microactuation in the final 3D structure due to the hierarchical order. We also envision the use of 3D structures composed of lamellar structures alternating conductive and non‐conductive domains with potential application in energy storage. Thus, we believe that the presented approach enabling the manufacturing of precise functional materials with defined nanostructure will open new opportunities in a wide range of application fields.

## 4 Experimental Section

### Chemicals and Materials

Chemicals and solvents were supplied from either Sigma–Aldrich or Fisher Scientific unless otherwise mentioned.

### Synthesis and Structure Characterization

To synthesize the block copolymers, the sequential RAFT polymerization was performed following an approach modified from Varadhajaran and Delaittre.^[^ ^36^ ^]^ The synthetic details are provided in the Supporting Information.

All synthesized compounds were characterized with ^1^H nuclear magnetic resonance (NMR) spectroscopy (Bruker Avance III 300, Bruker Avance III 600, or Bruker Avance Neo 700). GPC measurements were performed on a Shimadzu Nexera LC‐40 system (with LC‐40D pump, autosampler SIL‐40C, DGU‐403 (degasser), CBM‐40 (controlling unit), column oven CTO‐40C, UV‐detector SPD40, and RI‐detector RID‐20A).

The system was equipped with 4 analytical GPC‐columns (PSS): 1 × SDV precolumn 3 µm 8×50 mm, 2 × SDV column 3 µm 1000Å 8 × 300 mm, 1 × SDV column 3 µm 10e4Å 8 × 300 mm.

The measurements were performed in THF at a flow speed of 1 mL min^−1^ at a temperature of 40 °C. Chromatograms were analyzed using the LabSolutions (Shimadzu) software. Calibration was performed against different polymethylmethacrylate standards (800 – 2 200 000 Da, PSS) or polystyrene standards (370 – 2 520 000 Da, PSS).

### BCP Ink Preparation

A stock solution of DETC in 1,4‐dioxane, DMAc, or THF was added to give a final concentration of 0.25 wt.% photoinitiator and 30–60% BCP. The mixture was repeatedly mixed with a fine needle followed by centrifugation. Afterward, the mixture was agitated on an orbital shaker for 5 h followed prior to printing. The sample preparation was carried out under yellow light conditions.

### Silanization Procedure

Glass coverslips (Marienfeld, 170 ± 5 µm) were washed with isopropanol and acetone and dried using pressurized N_2_. Subsequently, the surface was activated for 5 min by plasma treatment. The coverslips were immersed in a 4 × 10^−3^ m solution of 3‐(trimethoxysilyl)propyl methacrylate in toluene for 1.5 h. After washing twice in toluene and once in acetone, the methacrylate‐functionalized glass slides were used for TPLP microfabrication.

### Two‐Photon Laser Printing of 3D Microstructures

Viscous ink (5–10 mg) was transferred to a silanized cover slide (22 × 22 mm, 170 µm thickness) with a spatula and covered with a smaller circular slide, which was gently pressed on to ensure good contact between the ink and the glass slide and reduce solvent evaporation. Two‐photon laser printing was performed within a commercially available setup (Photonic Professional GT2, Nanoscribe GmbH & Co. KG) in an oil immersion configuration with a femtosecond laser (λ = 780 nm) focused by a 63x oil objective (NA = 1.4; WD = 190 µm; Zeiss). Employing Describe software (Nanoscribe), GWL files were generated from STL files of desired geometries and executed by the printer for 3D structure fabrication. Slicing was set to either 200 or 300 nm and hatching to 200 nm for all microgeometries. Printing was performed with a varied scan speed of from 2 to 2.5 mm s^−1^ and laser power in the range of 17.5 to 22.5 mW depending on the structure. For development, the cover slide was placed into the same solvent used for the ink preparation until all excess ink was dissolved.

### Ultramicrotome

Self‐assembled bulk films were cut into small pieces and embedded in Epon (prepared by mixing 26.2 g glycid ether 100, 14.8 g dodecenylsuccinic acid anhydride, 9.2 g methyl‐5‐norbornene‐2,3‐dicarboxylic anhydride and 1.2 g benzyldimethylamine; all chemicals purchased from SERVA) and polymerized for 2 days at 62 °C.

Printed samples were stained in OsO_4_ (2% in Acetone) overnight, consecutively incubated in 50% and 70% solutions of Epon in acetone for 2–3 h, and then embedded in 100% Epon following the same procedure as for the film samples. The cover slide was detached from the embedded sample by submerging it in liquid nitrogen.

The resin blocks were trimmed to expose the structures. Ultrathin (100–120 nm) cross sections were cut using a PowerTome PC ultramicrotome (RMC Boeckeler) and placed on pieces of silicon wafer.

### Scanning Electron Microscope

The sections were imaged in a field‐emission scanning electron microscope (Ultra 55, Carl Zeiss Microscopy) at a primary electron energy of 1.5 keV, either with or without post‐staining in RuO_4_ vapor (0.5% in H_2_O, Polysciences Inc) for 30 min.

Images of entire 3D‐printed structures were obtained after sputter coating the sample with a 10–12 nm layer of Pt:Pd (80/20) using a field‐emission scanning electron microscope (Ultra 55, Carl Zeiss Microscopy) at a primary electron energy of 3 keV.

### SNOM

The sections were imaged in a commercial infrared scanning near‐field optical microscope (neaSNOM, Neaspec GmbH). Near‐field optical amplitude and phase images together with the topography of the sample (1 × 1 µm^2^ or 2.5 × 2.5 µm^2^, at least 200 × 200 pixels and 6.6 ms integration time) were acquired simultaneously by operating the system in tapping mode employing a pseudo‐heterodyne detection scheme.^[^ ^37^ ^]^ A platinum‐iridium coated probe (Arrow‐NCPt) with a resonance frequency of about 280 kHz was used and illuminated with a mid‐infrared quantum cascade laser (Daylight solutions). The laser was optimized using a power setting of 1.5 mW. The signal, which was detected with a mercury cadmium telluride detector, was demodulated at the third harmonic of the tapping frequency to suppress the background signal. By illuminating the sample at an independent absorption band of the methacrylate‐functionalized P(MMA‐*co*‐HEMA) at 1152 cm^−1^,^[^ ^38^ ^]^ it was possible to distinguish between the different block copolymer units and to image phase segregation via spectroscopic contrast. Regions with a high optical phase value could be assigned to P(MMA‐*co*‐HEMA)‐rich areas and those with low optical phase value to polystyrene‐rich areas.

### Small Angle X‐Ray Scattering

SAXS experiments were performed using a Xeuss 2.0 Q‐Xoom (Xenocs SA, Grenoble, France) instrument, equipped with a Genix3D Cu ULC (ultra‐low divergence) micro focus source of Cu Kα with an energy of 8.04 keV, a wavelength of 1.5406 Å, and a Pilatus3 R 300K detector (Dectris Ltd., Baden, Switzerland). The 3D printing ink was placed in a gel holder, packed between two polyimide foils with a sample thickness of 0.5 mm. Solid films were measured without substrate. The 2D scattering patterns were obtained using a sample‐to‐detector distance of 2500 mm, resulting in the range of accessible scattering vector (*q*) from 0.003 to 0.14 Å^−1^. An azimuthal integration of the scattering patterns was used to obtain 1D plots of the intensity *I*(q) versus *q* with *q* = 4πsin(2*θ*/2)/λ, where 2*θ* was the scattering angle and *λ* was the wavelength of the Cu Kα source. The measurement time was set to 600 s for both the film samples and the 3D printing inks.

## Conflict of Interest

The authors declare no conflict of interest.

## Supporting information

Supporting Information

## Data Availability

The data that support the findings of this study are available from the corresponding author upon reasonable request.
