# Supplementary material for: 3D Printing Hierarchically Nano‐Ordered Structures
Source: Adv Sci (Weinh). 2023 Aug 2;10(28):2302756. doi: 10.1002/advs.202302756 (PMC10558687; doi:10.1002/advs.202302756)
Supplement: Supplementary file 1 — Supporting Information [file ADVS-10-2302756-s001.pdf]

ADVANCED SCIENCE
Open Access

Supporting Information

for *Adv. Sci.*, DOI 10.1002/advs.202302756

3D Printing Hierarchically Nano-Ordered Structures

*Britta Weidinger, Guohui Yang, Nadine von Coelln, Hermann Nirschl, Irene Wacker, Petra Tegeder, Rasmus R. Schröder and Eva Blasco**

Supporting Information

3D Printing Hierarchically Nano-ordered Structures

*Britta Weidinger, Guohui Yang, Nadine von Coelln, Hermann Nirschl, Irene Wacker, Petra Tegeder, Rasmus R. Schröder, Eva Blasco**

Outline

1. Materials 2
2. Synthesis of block copolymers 2
   2.1 Synthesis of MacroCTA1: 2
   2.2 Exemplary synthesis of BCP1: 2
   2.3 Exemplary synthesis of BCP1a: 2
   2.4 GPC and ^{1}H NMR characterization 3
3. SEM and SNOM characterization of bulk films 7
4. SAXS characterization of bulk films and printing inks 8
5. Calculation of R_a using the Hansen solubility parameters 9
6. Characterization of 3D printed microstructures 10

1. Materials

Styrene, HEMA and MMA were passed over a column of basic alumina to remove the inhibitors. All other chemicals were used as received:

2. Synthesis of block copolymers (BCPs)

2.1 Synthesis of MacroCTA1:

In a Schlenk tube, MMA (6.40 g, 64.0 mmol, 819 eq.), HEMA (2.08 g, 16.0 mmol, 204 eq.) and CPDB (17.3 mg, 78.1 µmol, 1.0 eq.) were dissolved in 1,4-dioxane (8 mL). AIBN solution (323 mg, 9.78 µmol, 0.125 eq., 10 mg in 2.010 mg 1,4-dioxane) was added. The solution was degassed via freeze-pump-thaw (3 x 8 min), followed by backfilling with nitrogen. The reaction mixture was stirred at 90°C for 2.5 h, cooled in liquid nitrogen and opened to the atmosphere. After dilution with DCM, the solution was precipitated into diethyl ether twice. After centrifugation and decanting of the supernatant, copolymer macroCTA 1 was received as a light pink solid.

^{1}H NMR 600 MHz (CD_2Cl_2): δ [ppm] = 7.80 (m), 7.47 (m), 7.31 (m), 4.0 (bs), 3.74 (bs), 3.50 (s), 2.00 - 0.61 (m).

2.2 Exemplary synthesis of ***BCP1****:*

In a Schlenk tube, 560 mg (MW = 37641, 0.0149 mmol, 1 eq.) of macroCTA 1 were dissolved in 18 mL 1,4-dioxane. Styrene (18.3 g, 176 mmol, 11800 eq.) and AIBN stock solution (79 mg, 0.0032 mmol, 7.5 mg in 1.125 g 1,4-dioxane, 0.2 eq.) were added. The solution was degassed via freeze-pump-thaw (3 x 8 min), followed by backfilling with nitrogen. The reaction mixture was stirred at 90 C for 2 h, cooled in liquid nitrogen and opened to the atmosphere. The solvent and most of the remaining styrene were removed in vacuo, the viscous mixture was dissolved in small amounts of DCM and precipitated into diethyl ether twice. After centrifugation and decanting of the supernatant, BCP1 was received as a light pink solid.

^{1}H NMR 600 MHz (CD_2Cl_2): δ [ppm] = 7.80 (m), 7.47 (m), 7.31 (m), 7.28 - 6.31 (m), 4.07 (bs), 3.80 (bs), 3.56 (s), 2.14 - 0.63 (m).

2.3 Exemplary synthesis of ***BCP1a****:*

BCP1 (250 mg, MW = 93566, 2.67 µmol, 207 µmol OH-groups) was added to a Schlenk flask. After flushing with nitrogen, dry DCM (19 mL) was added. After dissolving, NEt_3 (592 µL, 430 mg, 4.24 mmol, 20 eq.) was added, the solution was cooled to 0 °C and methacryloyl chloride (348 µL, 389 mg, 3.74 mmol, 18 eq.) was added slowly under stirring. After stirring

at 0°C to RT for 16-72 h, most of the DCM was removed in vacuo. MeCN (65 mL) was added and the mixture was poured into $NaHCO_{3\ aq.}$(5%, 130 mL). After extracting with DCM (3 x 65 mL) the united organic fractions were dried over Na_2SO_4, the solvent was removed in vacuo and the washing step was repeated if NEt_3HCl was still present. After precipitation in Et_2O, FBCP 1 was obtained as a solid.

^{1}H NMR 600 MHz (CD_2Cl_2): δ [ppm] = 7.80 (m), 7.47 (m), 7.31 (m), 7.28 - 6.31 (m), 6.12 (bs), 5.63 (bs), 4.32 (bs), 4.17 (bs), 3.56 (s), 2.14 - 0.63 (m).

2.4 GPC and ^{1}H NMR characterization

Table S1: Composition, molecular weight, dispersity and molar fractions of the synthesized MacroCTAs.

Polymer		M_n (GPC)	Đ	f(HEMA)	Precursor for
MacroCTA1	P($HEMA_{93}$-MMA_{329})	37641	1.10	22%	BCP1, BCP2
MacroCTA2	P($HEMA_{50}$-MMA_{176})	23083	1.08	22%	BCP3
MacroCTA3	P($HEMA_{162}$-MMA_{138})	30000	1.08	46%	BCP4
MacroCTA4	P($HEMA_{69}$-MMA_{246})	33587	1.13	22%	

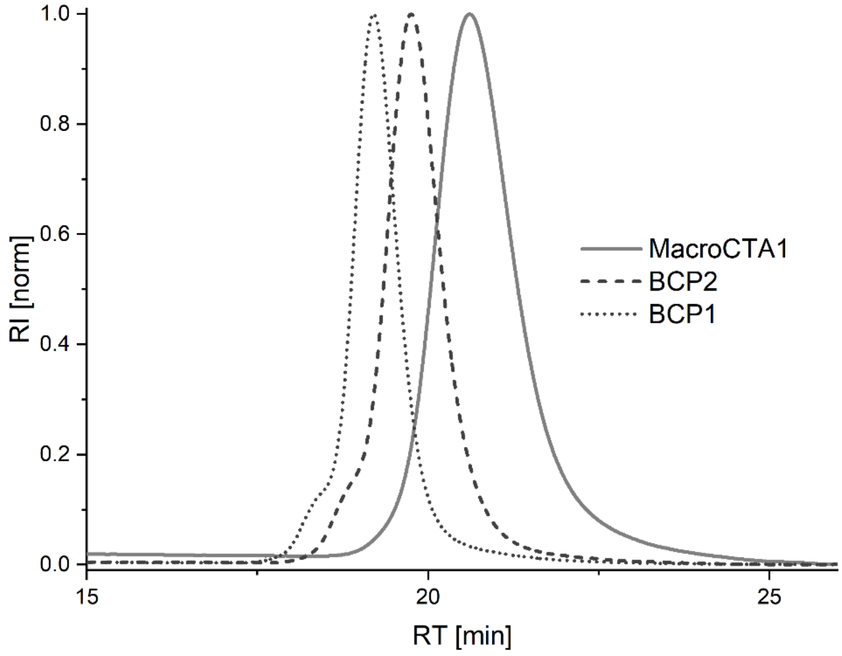

Figure S1: GPC traces of MacroCTA1, BCP1 and BCP2.

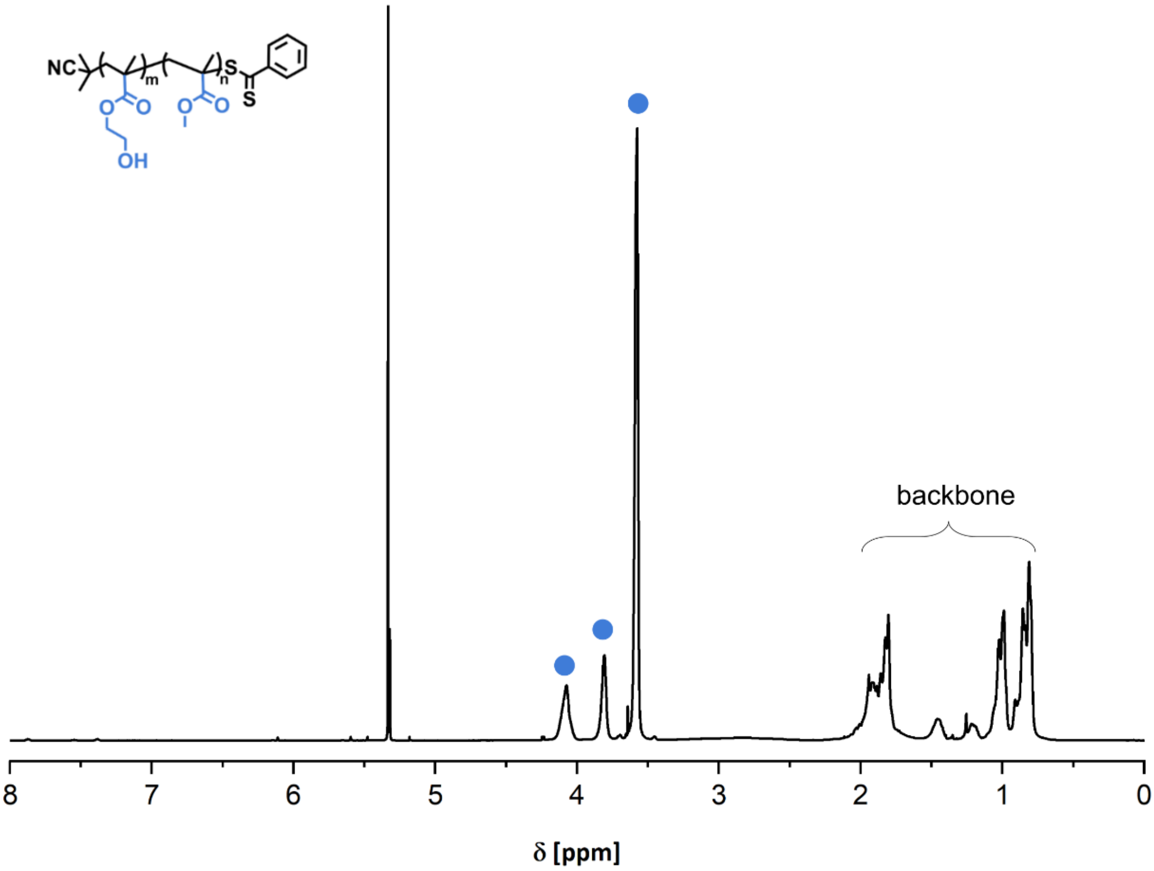

Figure S2: ^{1}H NMR spectrum of macroCTA1 in CH_2Cl_2.

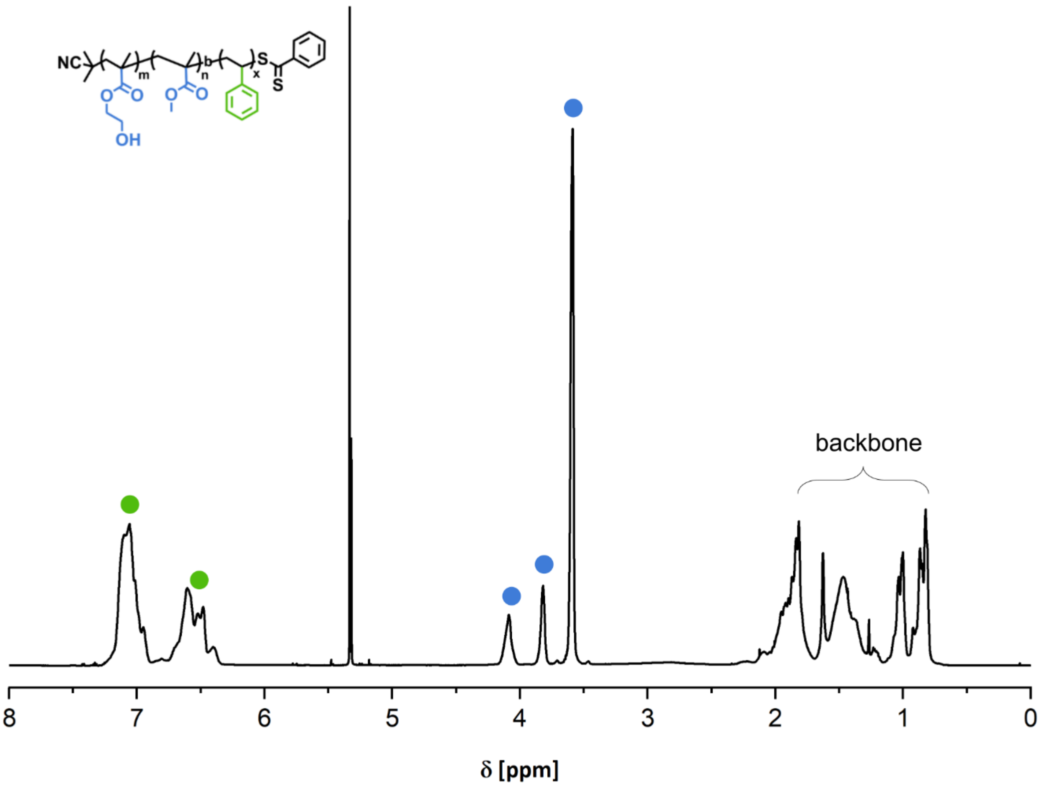

Figure S3: ^{1}H NMR spectrum of BCP1 in CH_2Cl_2.

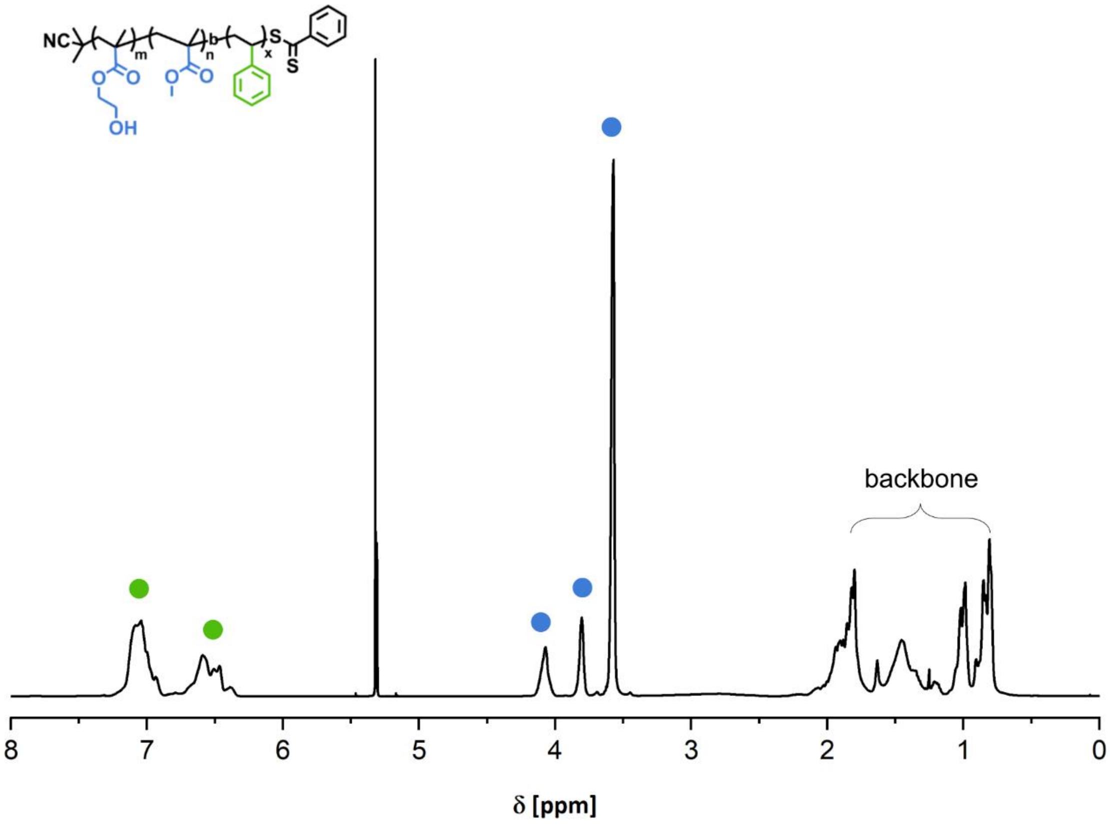

Figure S4: ^{1}H NMR spectrum of BCP2 in CH_2Cl_2.

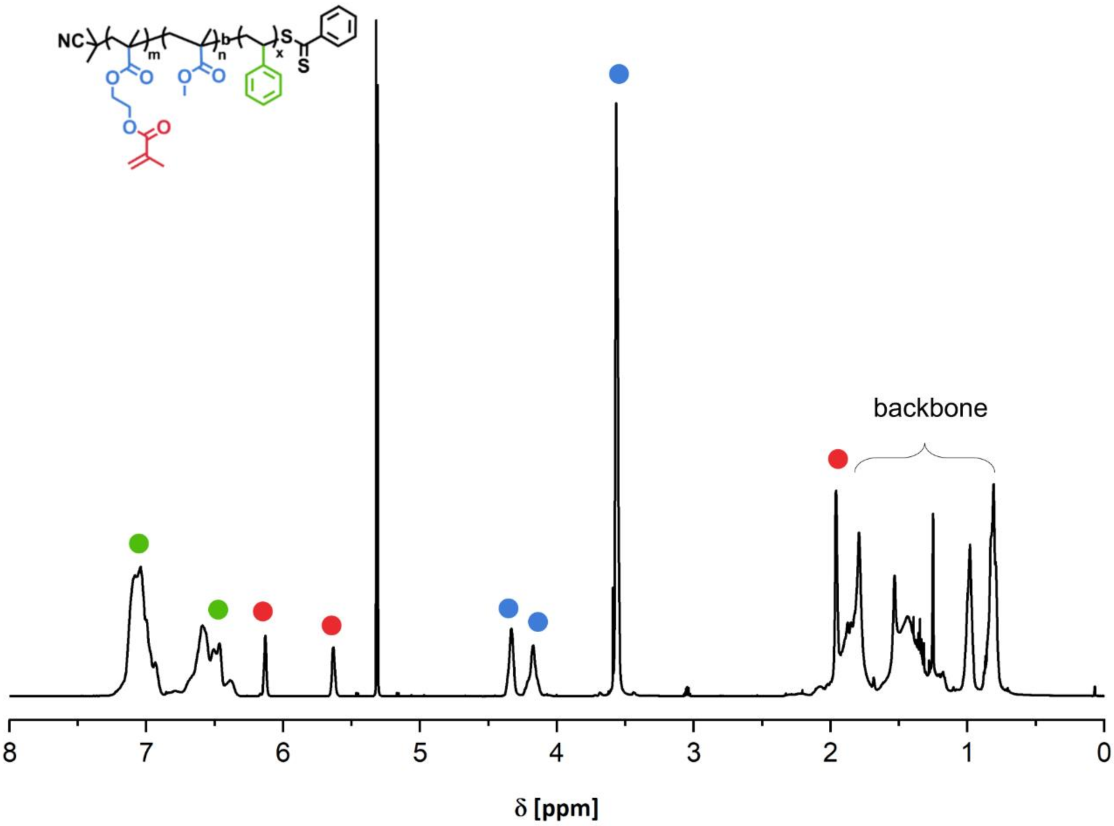

Figure S5: ^{1}H NMR spectrum of BCP1a in CH_2Cl_2.

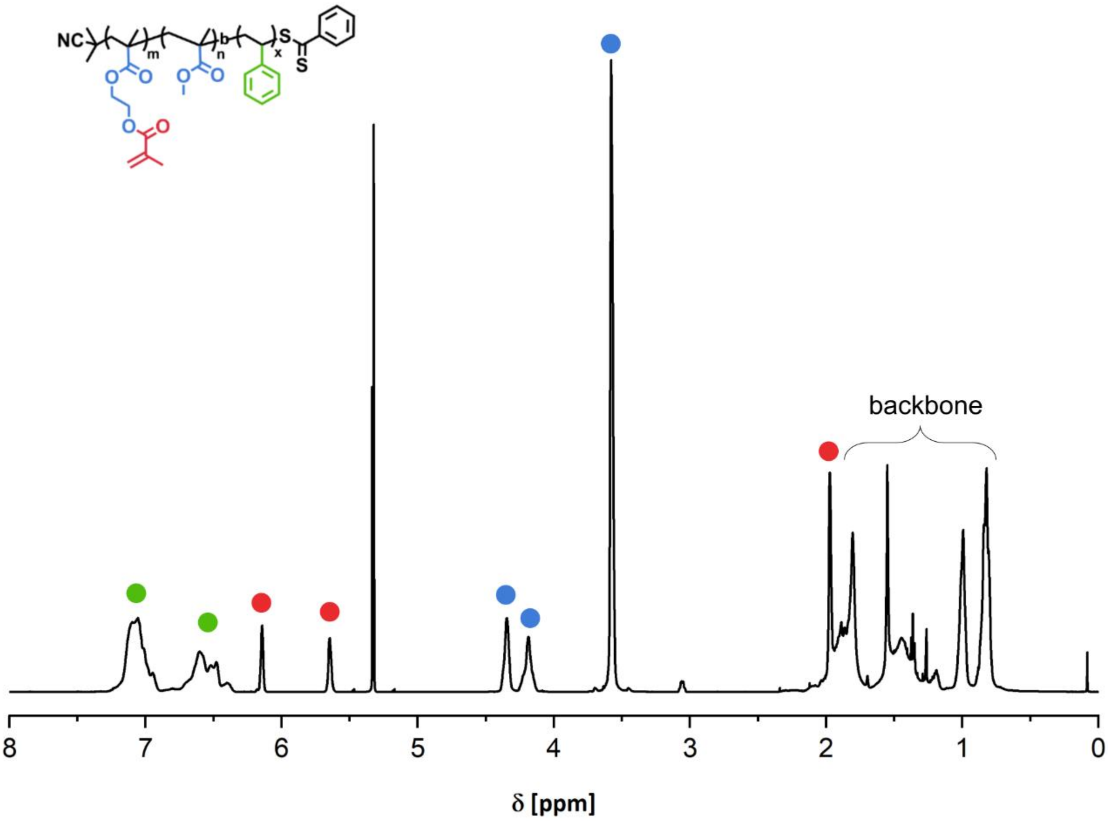

Figure S6: ^{1}H NMR spectrum of BCP2a in CH_2Cl_2.

3. SEM, SAXS and SNOM characterization of bulk films and printing inks

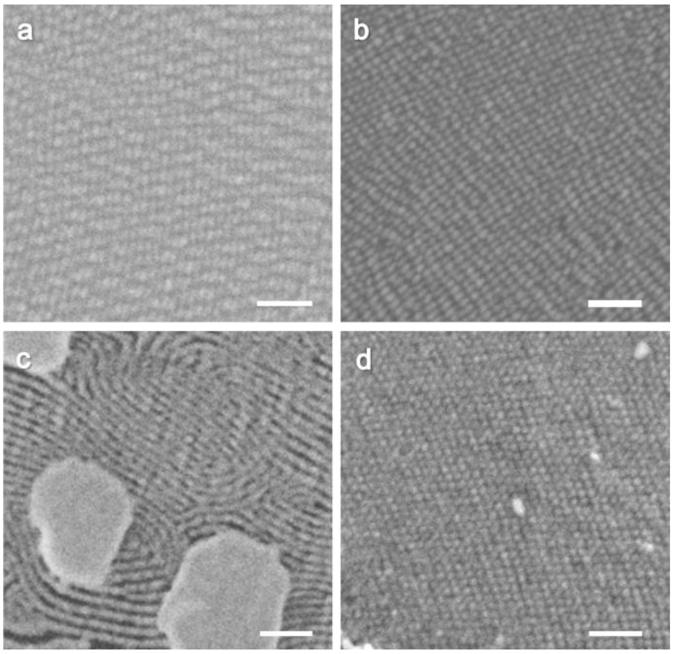

Figure S7: SEM images of cross-sections of block copolymer films a) BCP3, b) BCP3a, c) BCP4, d) BCP4a. Scale bars = 200 nm.

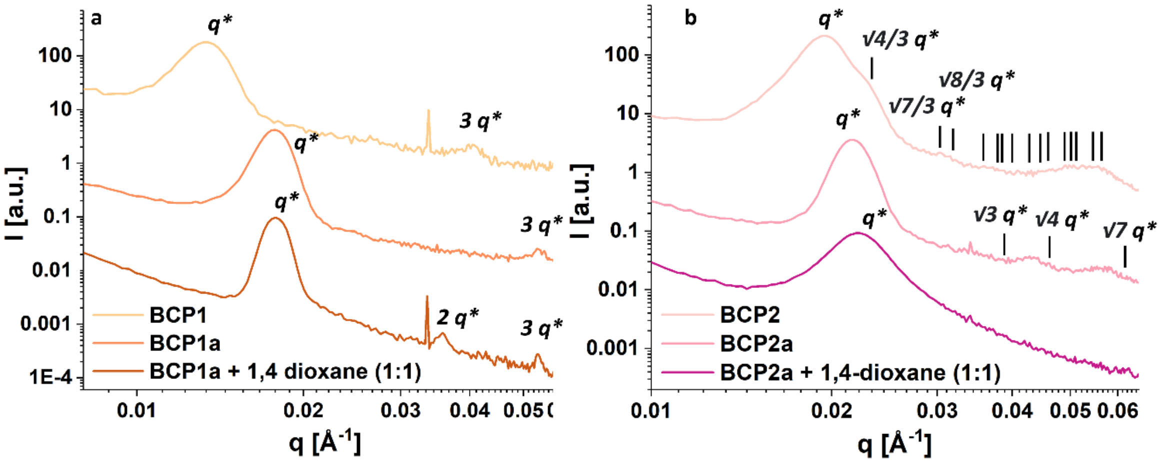

Figure S8: SAXS spectra of block copolymers, functionalized block copolymers and the ink compositions. Spectrum intensities are shifted for comparability. The q values used for assigning the morphologies are highlighted.

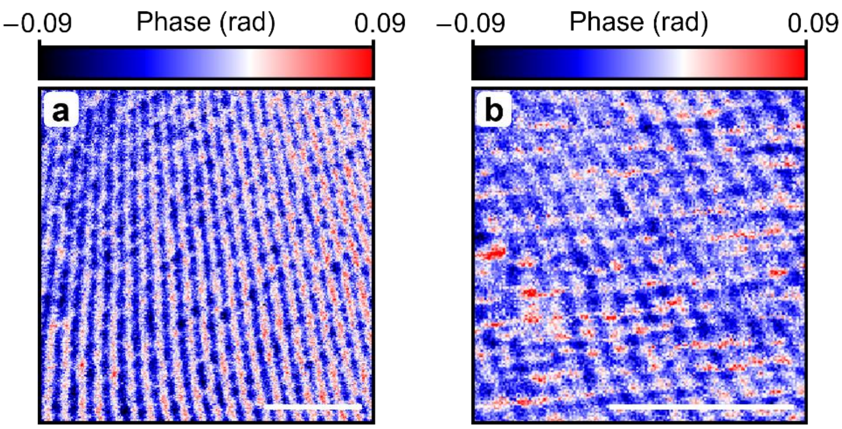

Figure S9: IR-SNOM analysis of cross-sectioned self-assembled films: a) near-field optical phase image mapped at 1152 cm^{-1} of BCP1a, b) near-field optical phase image mapped at 1152 cm^{-1} of BCP2a. Scale bars = 500 nm.

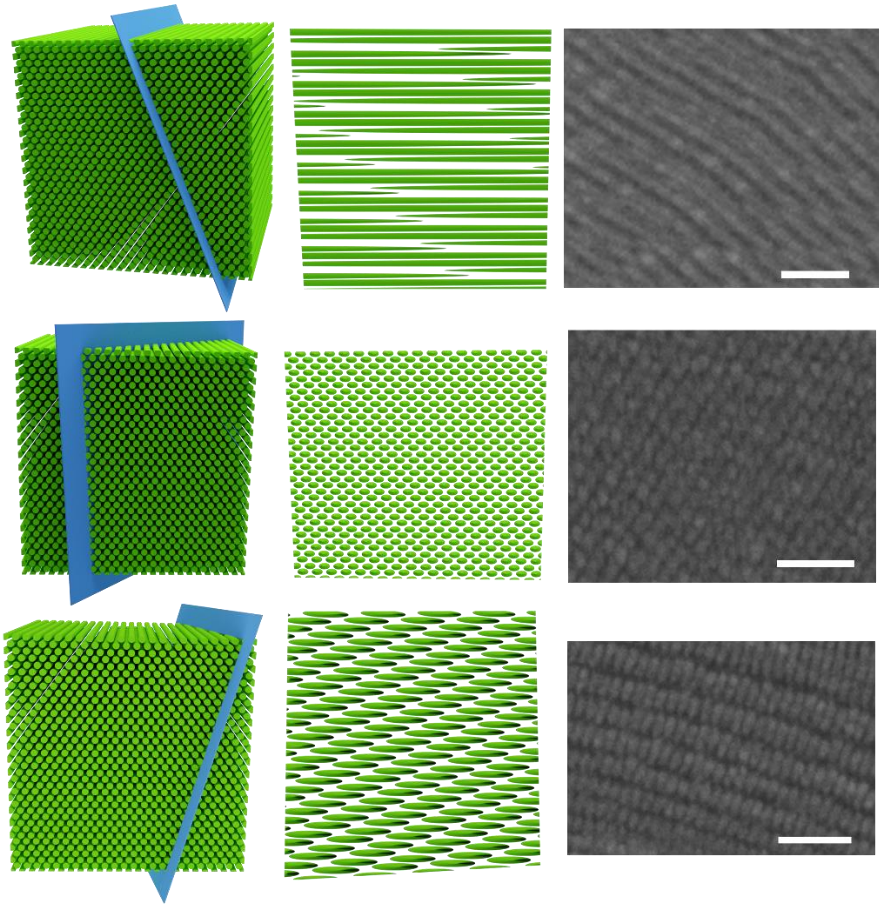

Figure S10: SEM images of cylindrical block copolymer BCP2a, in comparison with 3D modeled, rendered images cut along different planes of the ordered cylinders. Scale bar = 200 nm.

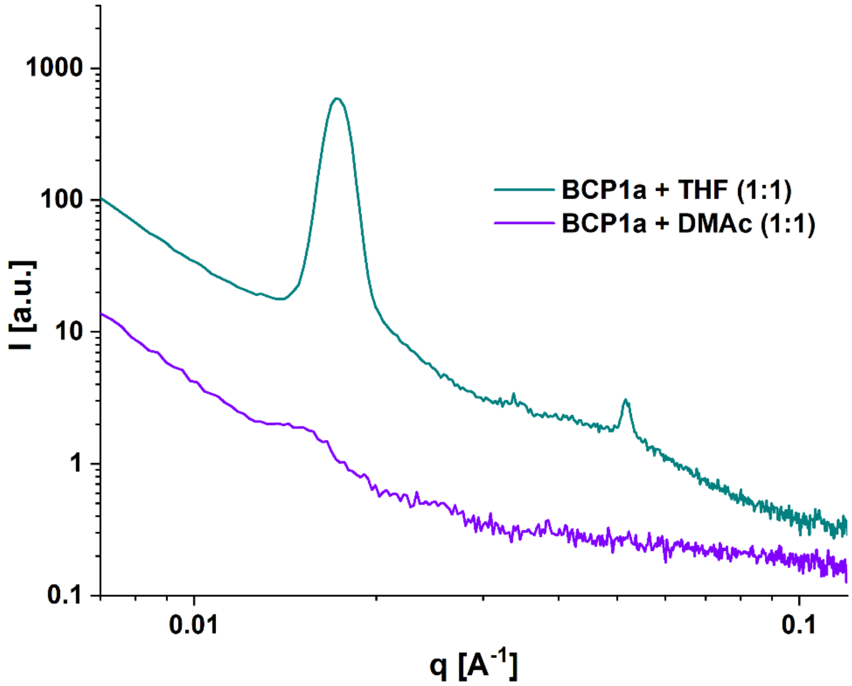

Figure S11: SAXS spectra of BCP1a in THF and DMAc, showing the lamellar order in THF and the weak ordering in the ink formulated with DMAc.

4. Calculation of R_a using the Hansen solubility parameters

Table S2: R_a calculated from the Hansen solubility parameters for the respective polymer and solvent using $R_a^2 = 4(\delta D1-\delta D2)^2 + (\delta P1-\delta P2)^2 + (\delta H1-\delta H2)^2$

Solvent	R_a (PS)	R_a (PMMA)
DMAc	10.7	6.3
THF	6.2	6.7
1,4-dioxane	7.0	9.8

5. Characterization of the 3D printed microstructures

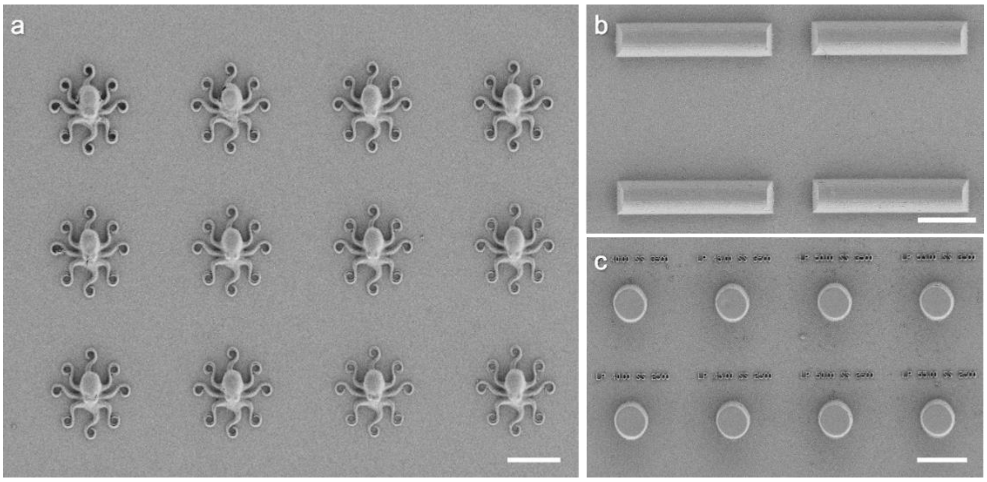

Figure S12: SEM images of 3D printed microstructures (a) octopi, b) half cylinders, c) pillars) using BCP1a and dioxane. Scale bar = 30 µm.

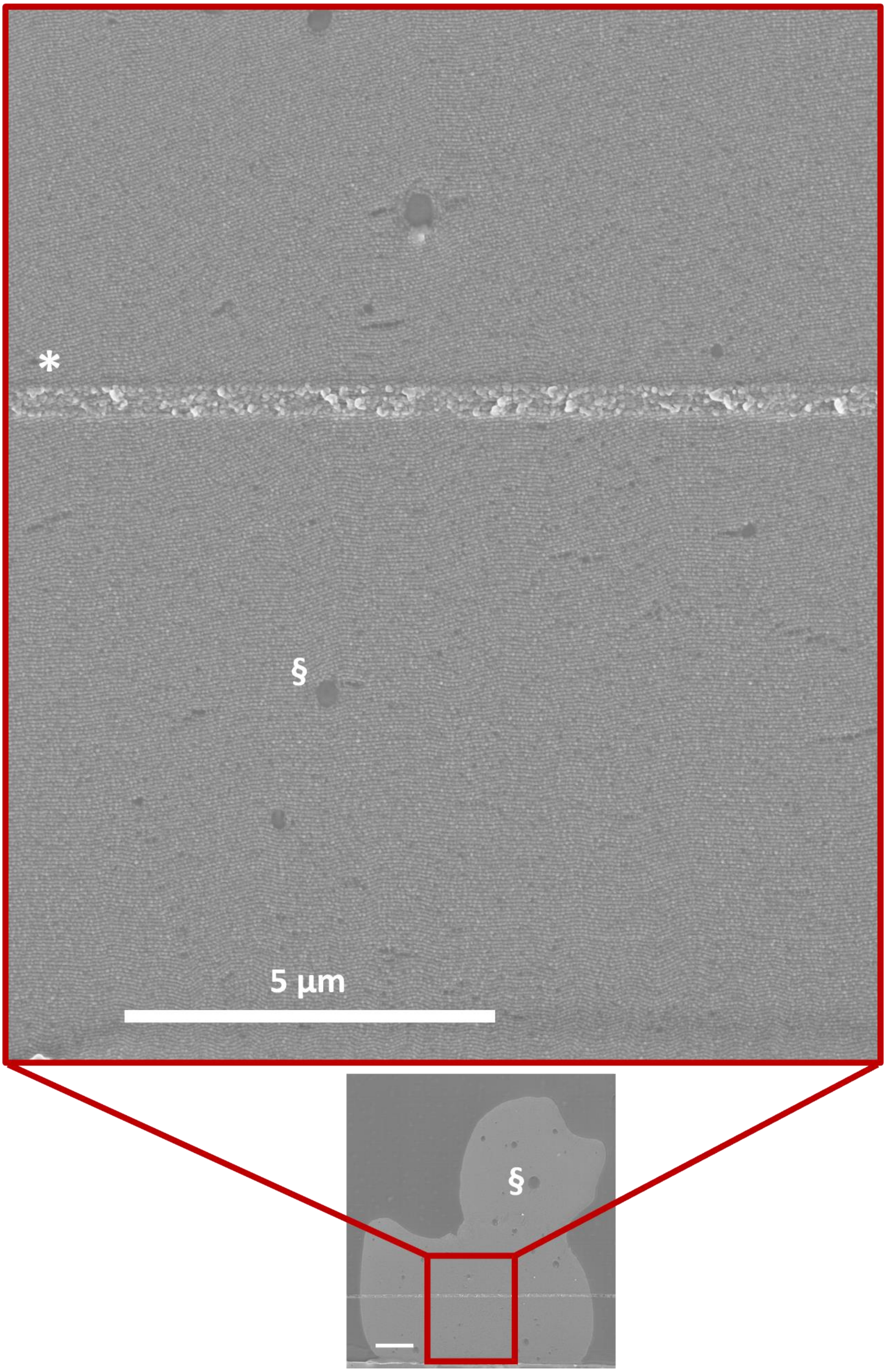

Figure S13: Zoom-in on the cross-section of a 3D microprinted duck using BCP1a. * indicates a knife mark from processing with the ultramicrotome whereas § indicates a small defect introduced by handling of the printing ink. A high resolution .tif image of the cross-section is available under https://doi.org/10.11588/data/IV5C94.

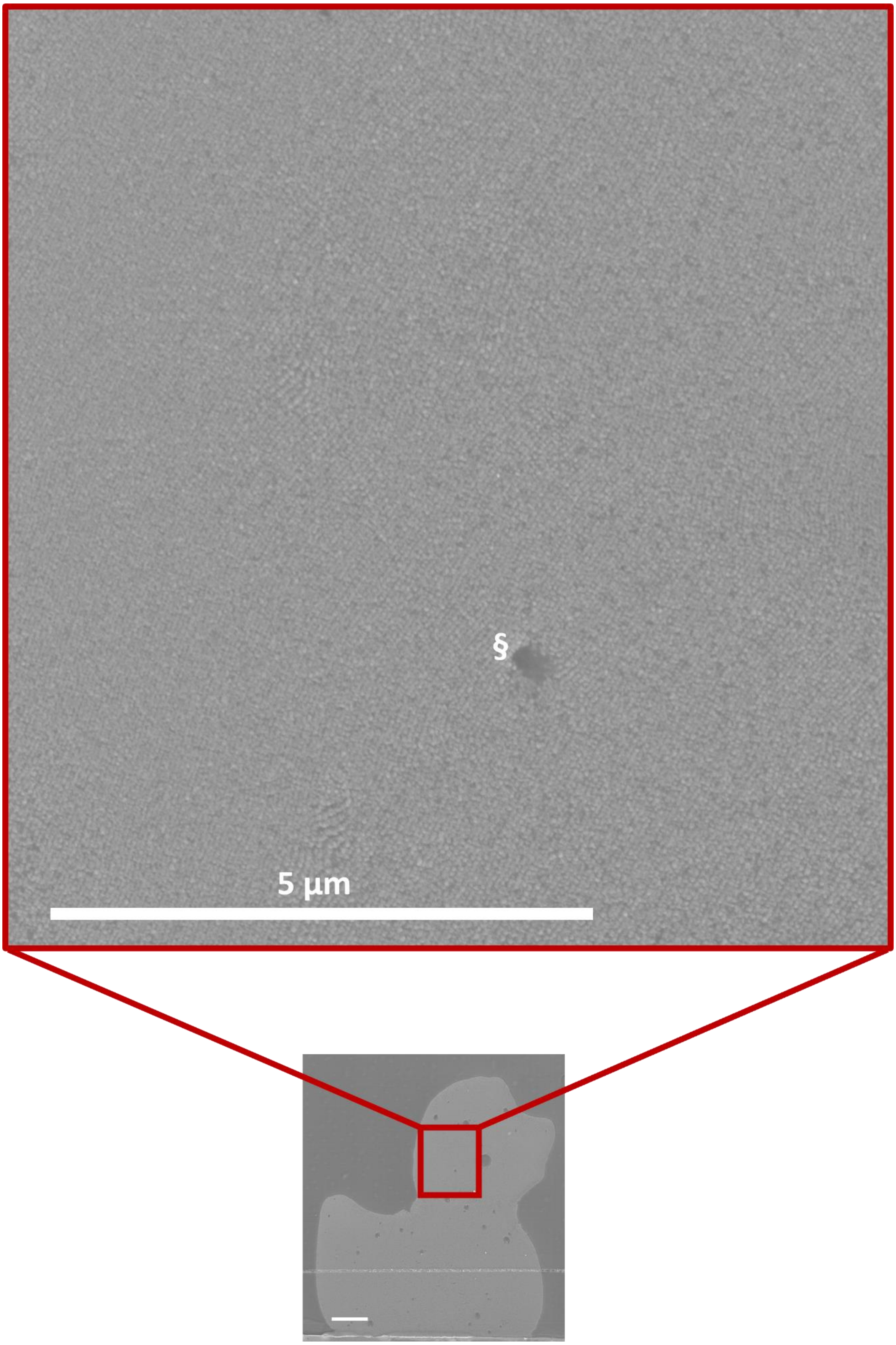

Figure S24: Zoom-in on the cross-section of a 3D microprinted duck using BCP1a. § indicates a small defect introduced by handling of the printing ink. A high resolution .tif image of the cross-section is available under https://doi.org/10.11588/data/IV5C94.

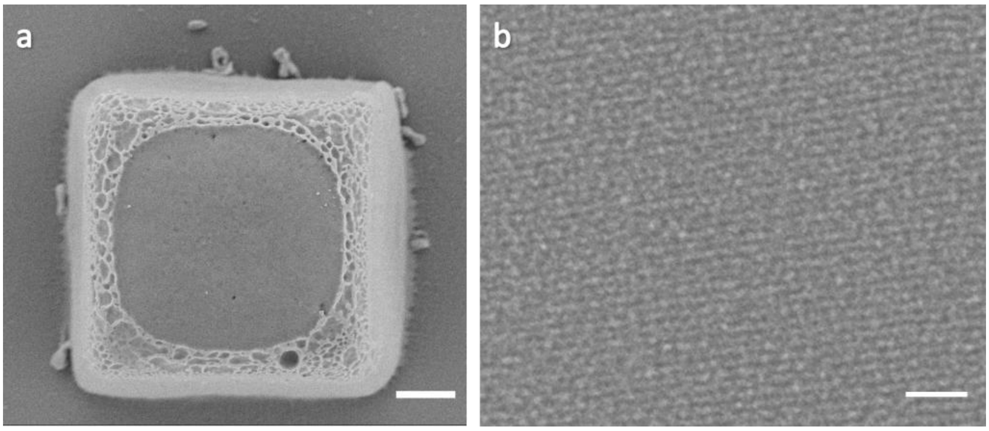

Figure S15: SEM image of microstructures printed using BCP1a and THF, a) of the entire structure, showing large defects in the top layer, b) in the cross-section. Scale bar left = 2 µm, right = 200 nm.

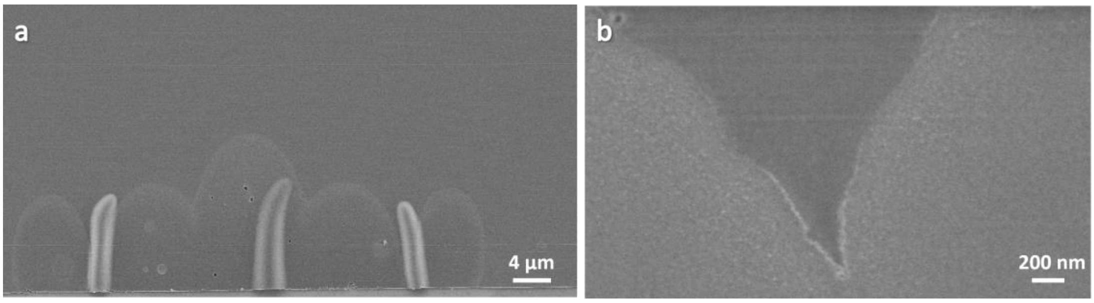

Figure S16: Cross-section (a) and zoomed in area (b) of a microstructure printed with functionalized P(MMA-*co*-AcryEMA) polymer in 1,4-dioxane as control sample.
